# Downregulating miR-432-5p exacerbates adriamycin-induced cardiotoxicity via activating the RTN3 signaling pathway

**DOI:** 10.18632/aging.206062

**Published:** 2024-08-22

**Authors:** Wei Geng, Shaohua Yan, Dasen Sang, Jie Tao, Xuefei Zhang, Xinshun Gu, Xiangyu Zhang

**Affiliations:** 1 Department of Cardiology, Baoding No.1 Central Hospital, Baoding, Hebei Province, China; 2 Department of Cardiology, The Second Hospital of Hebei Medical University, Shijiazhuang, Hebei Province, China

**Keywords:** adriamycin, cardiotoxicity, miR-432-5p, RTN3, LC3B, Beclin 1, cleaved caspase 3

## Abstract

Background: Adriamycin (ADR) is a widely used chemotherapy drug in clinical practice and it causes toxicity in the myocardium affecting its clinical use. miR-432-5p is a miRNA primarily expressed in myocardial cells and has a protective effect in the myocardium. We aim to explore the protective effect of miR-432-5p on ADR-caused impaired mitochondrial ATP metabolism and endoplasmic reticulum stress (ERs).

Method: The primary cardiomyocytes were obtained from neonatal mice and the ADR was added to cells, meanwhile, a mice model was constructed through intravenous ADR challenge, and expression levels of miR-432-5p were examined. Subsequently, the miR-432-5p was introduced *in vitro* and *in vivo* to explore its effect on the activity of mitochondrial ATP synthesis, autophagy, and ER stress. The bioinformatics analysis was performed to explore the target of miR-432-5p.

Results: ADR decreased the expression of miR-432-5p in cardiomyocytes. It also decreases mitochondrial ATP production and activates the ER stress pathway by increasing the expression of LC3B, Beclin 1, cleaved caspase 3, and induces cardiac toxicity. miR-432-5p exogenous supplementation can reduce the cardiotoxicity caused by ADR, and its protective effect on cardiomyocytes depends on the down-regulation of the RTN3 signaling pathway in ER.

Conclusion: ADR can induce the low expression of miR-432-5p, and activate the RTN3 pathway in ER, increase the expression of LC3B, Beclin 1, cleaved caspase 3, CHOP, and RTN3, and induce cardiac toxicity.

## INTRODUCTION

Adriamycin (ADR), or doxorubicin belongs to the anthracycline antibiotic family. ADR has a pharmacological effect on embedding with human DNA, thereby blocking tumor cell proliferation, and inducing cell apoptosis. It is a widely used broad-spectrum anti-tumor drug in clinical practice. Unfortunately, the side effects of ADR cardiotoxicity, such as typical dilated cardiomyopathy with increased cardiomyocyte apoptosis, can lead to heart failure in severe cases, limiting its clinical application [1]. The cause of cardiac toxicity by ADR is not fully understood and some progress has been made in its mechanism. The reported mechanisms include oxidative stress injury, cell apoptosis, mitochondrial damage, rapid degradation of cardiac actin, and autophagy damage caused by lysosomal damage [2–4].

The endoplasmic reticulum (ER) is the main site within cells for protein processing and synthesizing, calcium ions storing, and lipid and carbohydrate metabolism [5]. When cells experience stress, such as hypoxia, nutrient deficiency, calcium homeostasis imbalance, etc., the ER function is disturbed, and causes unfolded or misfolded proteins to accumulate in the ER cavity, leading to a pathological state of ER dysfunction, known as endoplasmic reticulum stress (ERs) [5, 6]. To alleviate ER stress, cells initiate an unfolded protein response (UPR) and reduce unfolded or misfolded proteins by activating molecular chaperone expression, regulating lipid synthesis, promoting ER-related degradation, restoring ER homeostasis, and improving cell survival ability under adverse conditions. When it undergoes stress responses, the body adapts to cope with stress at the organelle level. The endoplasmic reticulum and mitochondria are important subcellular organelles involved, and the two are interconnected. ERs and mitochondrial dysfunction are correlated with myocardial injury [7].

The myocardial cells have limited proliferative capacity, they have higher proteasome activity and higher protein turnover compared to other tissues. The unfolded protein response induced by endoplasmic reticulum stress leads to an apoptotic cascade, and the cardiotoxicity of ADR is related to the sustained activation of unfolded protein response leading to myocardial cell apoptosis. Reticulon3 (RTN3) is a member of the RTN family primarily located in the endoplasmic reticulum. Research has demonstrated that RTN3 is widely expressed in multiple tissues and organs, and is mainly distributed in the ER, Golgi apparatus, plasma membrane, and extracellular space [8–10]. RTN3 has apoptosis functions. Overexpression of RTN3 mediates the three major apoptosis signaling pathways in eukaryotic cells [11]. Elevated RTN3 has been reported in the myocardium of patients with heart failure after myocardial infarction [12]. Moreover, Reticulon 3 deficiency ameliorates post-myocardial infarction heart failure in myocardial infarction mice [12]. In lipid droplet accumulated cardiac dysfunction, RTN3 assisted the transport of fatty acids to the ER [13]. These results indicate an increase in RTN3 during the process of myocardial cell damage. The roles of RTN3 on cardiac toxicity caused by ADR are not clear. In the current study, we established *in vivo* and *in vitro* models to study the role of miR-432-5p and RTN3 in cardiac toxicity caused by ADR.

## MATERIALS AND METHODS

### Ethical approval

The animal study was approved by the animal ethic committee of Baoding No. 1 Central Hospital, and all protocols followed the guideline of the Institutional Animal Care and Use Committee (approval number: K-2023-145). C57BL/6 male mice aged 6–8 weeks were used for *in vivo* analysis, and two ADR doses (#E2516, Selleck, USA, 5 mg/kg and 7.5 mg/kg) were single injected to tail vein of mice to induce cardiotoxicity in mice. The mice in the control group received physiological saline. Six mice were included in the final analysis. The survival status of mice was observed, and the body weight was recorded every 2 days after injection. All mice were euthanized by decapitation after 14 days of injection for biochemical and pathological analysis.

### Cardiac function measure

The cardiac function measurements were performed by the Vevo 770 ultrasound system (Visualistics Inc., Toronto, Canada) and was operated by an ultrasound medical professional. The left ventricular ejection fraction (LVEF) and left ventricular systolic function (LVSF) in each group were measured according to the provided instructions and reports [14].

### Hematoxylin and eosin (H&E) staining and semi-quantify analysis

The mice were anesthetized by carbon dioxide following the method reported previously [15]. The abdominal cavity of mice was exposed after disinfection. The heart was quickly harvested and fixed by 95% ethanol and glacial acetone for 24 hours. The tissue was subjected to gradient dehydration and immersed in paraffin to prepare 6 μm tissue slices. The slice was dewaxed, and antigens were repaired. Slices were stained with Harris hematoxylin for 5 minutes, washed with water, differentiated with 1% hydrochloric acid alcohol for 5 seconds, and then colored with 0.6% ammonia. Finally, the slices were immersed in an eosin staining solution for 2 minutes and then dehydrated and sealed with neutral gum. The slides were observed under a microscope, and the images were captured. Following the methods reported by Oei et al. [16], the injury score of myocardial tissue was analyzed. The criteria for the score include contraction bands (0, 1, 2, and 3); interstitial edema (0 and 1); interstitial hemorrhage (0, 1, and 2); platelet aggregates (0 and 1); and extravasation of red blood cells (0 and 1).

### Mitochondrial ATP synthesis detection

The ATPase activity is an important marker of mitochondrial activity or injury state. ATPase activity was measured by a commercial kit (#BC0065, Solarbio Inc., Beijing, China) after mice were sacrificed immediately. A total of 0.1 g of myocardium was homogenized in the ice bath and then centrifuged with 8000 g at 4°C for 10 minutes. The supernatant was kept and placed on ice. The tested sample was loaded in a 96-well plate, and the light absorption value at 660 nm on the microplate reader was recorded to calculate enzyme activity. A 10 μmol/mL standard phosphorus stock solution was prepared. One enzyme activity unit (IU) is considered as ATPase decomposes ATP and produces 1 μmol of inorganic phosphorus from 1 per milligram of tissue per hour. The final concentration of the tested sample was presented as IU/L.

### Primary cardiomyocytes isolation

We isolated primary cardiomyocytes from newborn C57BL/6 mice following the methods reported by Ravi et al. [17]. Briefly, newborn mice were euthanized by decapitation with a sharp pair of scissors, and the heart was squeezed to dissect the left ventricle. The stripped tissue was immersed in pre-cooled PBS solution and cut into pieces. After digested by 0.05% collagenase and 0.05% trypsin, the tissue was centrifuged at 1500 rpm for 5 minutes. The lower layer of centrifuged solution was cultured in DMEM-F12 medium (11320033, Gibco, USA) containing 15% FBS in the tissue culture dish. After 2 hours of cultivation, the unadhered cells containing cardiomyocytes in the medium were collected. The cell suspension was centrifuged at 2000 rpm for 5 minutes, resuspended in MEM-F12 medium and cultivated for further analysis after counting numbers under an optical microscope (Nikon Company, Japan). All measured experiments were performed in triplicate.

### Plasmids

The has-miR-432-5p agomir (#miR40002814-4-5, Ribobio, Guangzhou, China) or scramble were diluted, and the *in vivo* introduction was performed 1 day after ADR stimulation. The agomir (5 nmol) was diluted by 200 microliters of physiological saline and injected through the tail vein in 5 consecutive doses for 5 days. For *in vitro* analysis, miR-432-5p mimic (#miR10002814-1-5, Ribobio), or si-RTN3 (#siG000010313C-1-5, Ribobio) or the full-length of RTN3 sequence (OE-RTN3, #MG51210-ACGLN, Sino Biological Inc., Beijing, China) was transfected to ADR stimulated cardiomyocytes by Lipofectamine 2000 (#11668027; Thermo Fisher Scientific, USA) regent following the manufacturer’s instruction [18] 2 days prior stimulation.

### Bioinformatics and luciferase reporter analysis

The potential binding targets of miR-432-5p were screened online databases including ENCORI, miRPathDB, TargetScan, miRWalk, and DIANA. To explore the involved function of genes, the obtained genes were subjected to the Database for Annotation, Visualization, and Integrated Discovery (DAVID) (https://david.ncifcrf.gov/) for further information analysis. The binding sites between miR-432-5p and RTN3 were predicted by the above database. According to the sequence reported in PubMed (https://www.ncbi.nlm.nih.gov/nuccore/NM_006054.4), the fragment containing the binding site (human RTN3 513-519aa) or mutant sequence (RTN3-WT, RTN3-Mut) of miR-432-5p was inserted into pGL3-basic (#E1751, Promega, USA) vector by XhoI and BglII sites for luciferase reporter analysis. The vectors were co-transfected with vectors harboring miR-432-5p mimics or negative sequence (NC) into HEK-293 cells over 80% confluence by Lipofectamine 2000 regent (#11668019, Invitrogen, USA). The firefly luciferase activity was analyzed by a luciferase assay system (#E1500, Promega) and calculated as the relative activity of cells transfected with miR-432-5p NC fragment.

### CCK-8 assay

The viability of cardiomyocytes after two doses of ADR stimulation (0.5, 1.0, 2.0, and 4.0 μM) was measured. Cells in the control group received solvent (H_2_O) of ADR. The cells were inoculated in 96-well plates (1 × 10^4^ cells/well) at 37°C and cultured in 5% CO_2_ for 24 h. Ten μl CCK-8 solution (#C0042, Beyotime, Shanghai, China) was then added to each well and cultured for another 2 hours. The absorbance value of each well at 450 nm was measured by a microplate reader. The viability of cells was calculated as the percentage of the control group.

### Reactive oxygen species (ROS), intracellular interleukin 1 beta (IL-1β) and lactate dehydrogenase (LDH) measurement

After stimulated by ADR, cells lysis regent (P0013, Beyotime, China) was put on ice for 30 minutes. The lysed solution was centrifuged for 12000 r/min for 15 minutes in 4°C, and the supernatant was collected to measure the ROS level (#CA1410, Solarbio Inc.), intracellular IL-1β (#SEKM-0002, Solarbio Inc.) and LDH (#BC0685, Solarbio Inc.) according to the protocols provided by the manufacturer.

### Quantitative reverse transcription PCR (qRT-PCR)

The qRT-PCR was adopted to measure target genes. After separation by TRIzol reagent (#15596026, Invitrogen, USA), the total RNA was extracted and determined the integrity of the obtained RNA. Total RNA was reverse transcribed into cDNA by a two-step real-time RT-PCR kit (RR037A, TaKaRa, Japan) in a 10 μl reaction. The SYBR Green detection (RR420, TaKaRa, 20 μl reaction mixture) was adopted to perform RT-PCR in Applied Biosystems^®^ 7300 system (Foster City, CA, USA). The relative expression of targeted genes was calculated by 2^−ΔΔCt^ method. The U6 RNA or GAPDH were adopted as internal references. The primer for RT-PCR was listed in Table 1.

**Table 1:** The primers for RT-PCR and sequences.

**Gene**	**Primer sequence (from 5′ to 3′)**
miR-432-5p forward:	GCTCTTGGAGTAGGTCATTGGGTG
miR-432-5p reverse:	TGGTGTCGTGGAGTCG
U6 forward:	CTCGCTTCGGCAGCACA
U6 reverse:	AACGCTTCACGAATTTGCGT
RTN3 forward:	CACAGGTAGAAATGGCCAAGA-
RTN3 reverse:	CAGCTTGAATGACAGACTTATAGACT-
GAPDH forward:	CATCACTGCCACCCAGAAGACTG
GAPDH reverse:	ATGCCAGTGAGCTTCCCGTTCAG

### Western blot

The total protein was extracted by protein lysate and homogenized thoroughly on ice. The homogenate is centrifuged at 12000 g for 15 minutes in a 4°C precooled centrifuge. The supernatant was transferred to a new Eppendorf tube, and the protein levels were quantified by BCA methods. Thirty micrograms of protein samples were loaded in well for sodium dodecyl-sulfate polyacrylamide gel electrophoresis (SDS-PAGE). After converted to polyvinylidene fluoride (PVDF) membrane. The nonspecific antigens were blocked by 5% skimmed milk powder for 2 hours, then the primary antibody for LC3B (#18725-1-AP, 1:500 dilution), cleaved caspase-3 (#ab214430, 1:5000 dilution, Abcam), Beclin 1 (#11306-1-AP, 1:2000 dilution, Proteintech), RTN3 (#12055-2-AP, 1:3000 dilution, Proteintech), CHOP (#66741-1-IG, 1:2000 dilution, Proteintech) were added and cultured in a 4°C refrigerator overnight. The secondary antibody was added to membrane and cultured for another 2 hours. The bands were developed by ELC reagent. The images were captured and analyzed by the Image Lab Software (Bio-Rad Laboratories, Hercules, CA, USA).

### Statistics

SPSS 20.0 statistical software was used for data analysis and data are presented by GraphPad 9.3 software. The student’s *t*-tests were used for the comparison between groups with normal distributed data. For multiple group comparison, one-way analysis of variance (ANOVA) was adopted and followed by Newman-Keuls post hoc analysis. *P* < 0.05 was statistically significant.

### Data availability

The analyzed data are available from the corresponding author upon reasonable request.

## RESULTS

### ADR results in cardiomyocyte damage and impairs cardiac function

**Figure 1:**
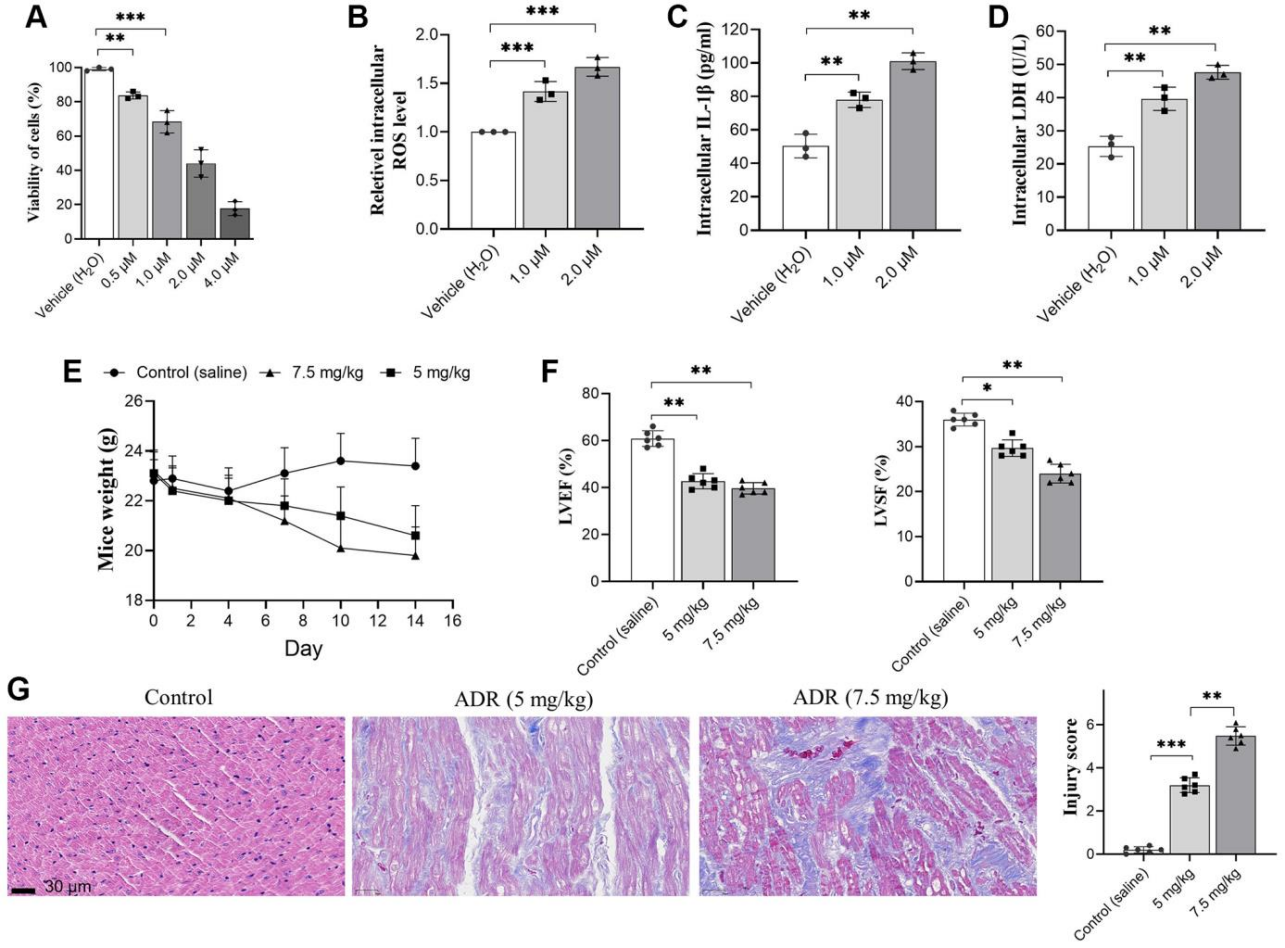
**Adriamycin (ADR) treatment damages cardiomyocyte and impaired cardiac function of C57BL/6 mice.** (**A**) Cardiomyocytes were treated by a serial of ADR, and ADR decreases the viability of cardiomyocytes; (**B**–**D**) ADR stimulates oxidative stress and inflammatory response. ADR causes increasing of intracellular reactive oxygen species (ROS) (**A**) and interleukin-1β (IL-β) (**B**) lactate dehydrogenase (LDH) (**C**) level, and LDH levels in primary cardiomyocytes (**D**); (**E**) ADR causes obvious decreasing of body weight, and impaired left ventricular ejection fraction (LVEF) and left ventricular systolic function (LVSF) in mice (**F**); (**G**) Hematoxylin and Eosin (H&E) staining in ADR-treated myocardium of mice. Increased collagen between muscle, irregular arrangement of myocardial cell nuclei, granular and interstitial hemorrhage could be observed. ^**^p < 0.01, ^***^p < 0.001.

The injury of ADR on primary cardiomyocytes was analyzed *in vitro* by CCK-8 assay. Our results showed that ADR inhibited the viability of cells significantly at serial concentrations (Figure 1A). Based on this results, two ADR concentration, 1.0 and 2.0 μM were selected for subsequent *in vitro* analysis. ER stress causes oxidative stress and apoptosis in myocardial tissues [19]. The ROS level in ADR-stimulated cells was measured by a commercial kit. We found that ADR increased intracellular ROS production in cells (Figure 1B), as well as the intracellular IL-1β levels (Figure 1C), and increased LDH levels (Figure 1D). We observed decreased body weight in both ADR-treated animal groups (Figure 1E). The LVEF and LVSF (Figure 1F) also decreased *in vivo*. By H&E immunohistochemical staining, we observed neatly arranged myocardial cells in control mice, with short columnar and branched cells. The nucleus is located in the center of the cell with an elliptical shape (Figure 1G). The myocardial tissue of the ADR-treated groups was obviously damaged with higher injury scores. We could observe the disordered arrangement of muscle fibers, myocardial interstitial hemorrhage and edema, and increased interstitial collagen deposition between muscle fibers and muscle bundles in some areas. Meanwhile, irregular arrangement of the myocardial cell nucleus, granular and fatty degeneration of cytoplasm, and occasional infiltration of inflammatory cells could also be observed (Figure 1G).

### ADR stimulation causes decreased miR-432-5p expression in myocardial cells and tissues

**Figure 2:**
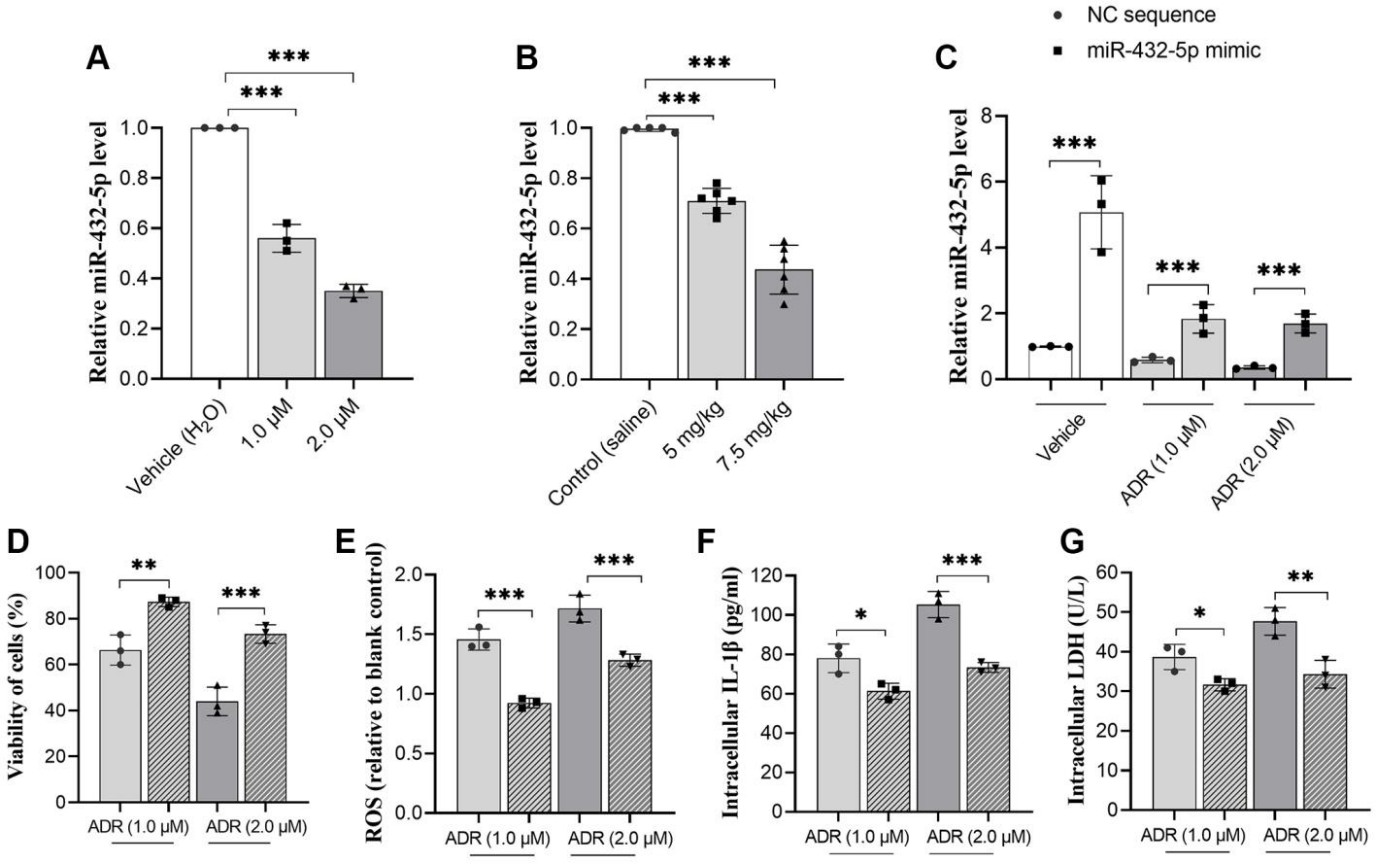
**miR-432-5p alleviates ADR-caused cardiomyocytes injury.** (**A**) The relative level of miR-432-5p in primary cardiomyocytes treated with ADR; (**B**) The relative level of miR-432-5p in ADR-treated mice; (**C**) The miR-432-5p mimic was transfected to primary cardiomyocytes, RT-PCR was performed to confirm the successful of transfection; (**D**–**G**) The increasing of miR-432-5p improved the cell viability caused by ADR; decreased ROS, IL-1β, and LDH levels in ADR treated cells. ^*^p < 0.05, ^**^p < 0.01, ^***^p < 0.001.

The changes of miR-432-5p in ADR-treated cells and tissues were measured. ADR causes decreased expression of miR-432-5p in primary cardiomyocytes and myocardium (Figure 2A, 2B). To clarify whether miR-432-5p mediates the changed function of cardiomyocyte cells. The vector containing miR-432-5p was introduced to cells and upregulated miR-432-5p was confirmed (Figure 2C) in ADR-treated cells. The CCK-8 analysis confirmed that after exogenous upregulation of miR-432-5p, the viability of cardiomyocytes increased significantly (Figure 2D). In addition, the decreased ROS (Figure 2E), intracellular IL-1β (Figure 2F) and decreased intracellular LDH levels (Figure 2G).

### RTN3 is activated in ADR-treated myocardial tissues, also a potential target for miR-432-5p

**Figure 3:**
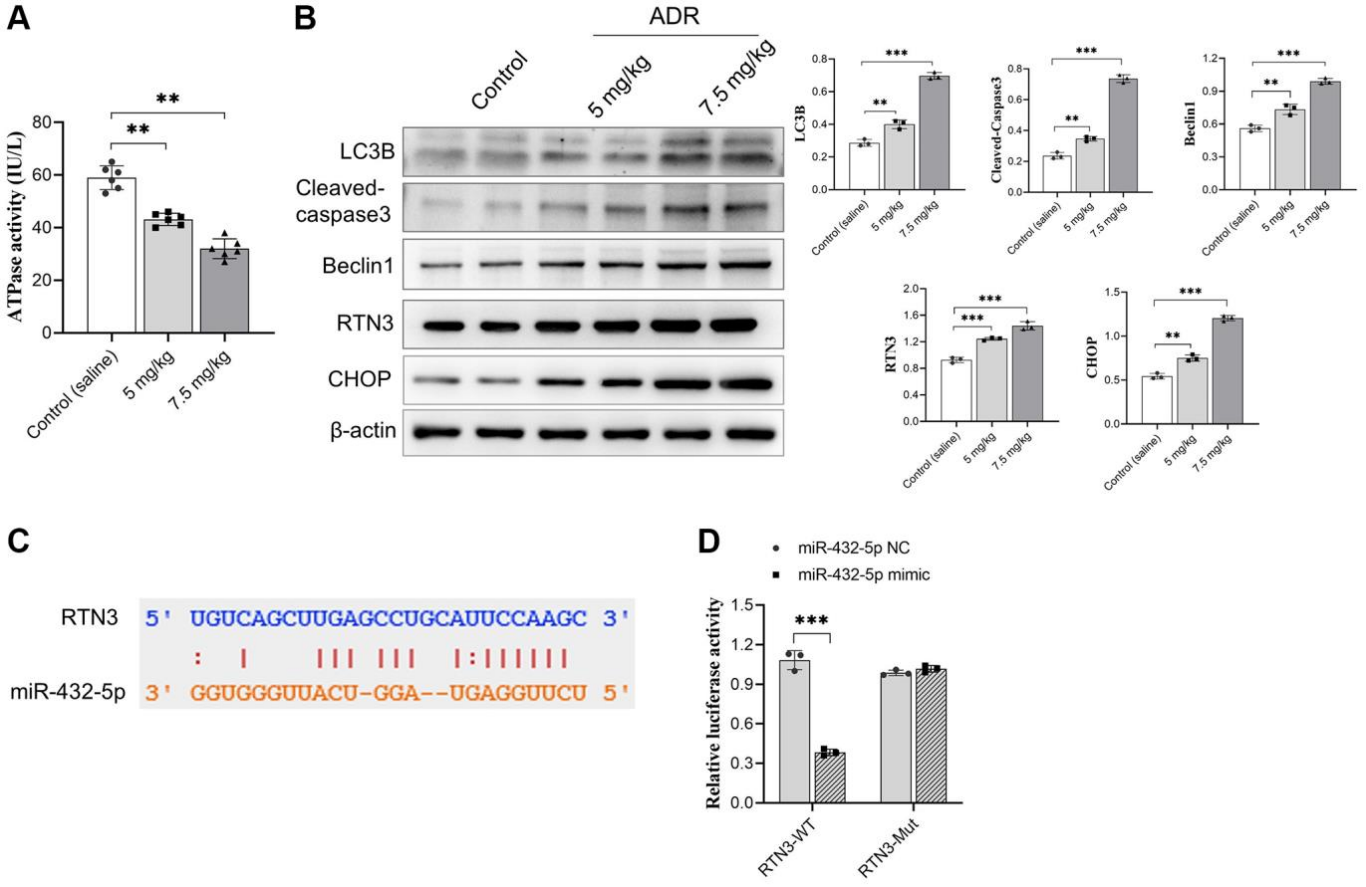
**RTN3 activation in ADR-treated cardiomyocytes.** (**A**) The decreasing ATPase activity in cardiomyocytes; (**B**) The expression of LC3B, cleaved-caspase 3, Beclin 1, RTN3, and CHOP protein increased in ADR-treated myocardium; (**C**) The binding site between RTN3 and miR-432-5p; (**D**) The binding of RTN3 and miR-432-5p is verified by luciferase report gene. ^**^p < 0.01, ^***^p < 0.001.

We previously reported that miR-432-5p has a protection effect on myocardial infarction through activating Nrf2 pathway by degrading Keap1 via direct interaction [20]. To further explore the possible regulating genes of miR-432-5p, the potential binding genes were screened again. We observed RTN3, a protein which is located into the ER membrane and has potential binding sites with miR-432-5p, and the binding sites was shown in Figure 3A. Next step, the direct binding between miR-432-5p and RTN3 was verified by dual luciferase assay (Figure 3B). Overexpression of RTN3 was reported in the heart injury and was correlated with ER stress, impairment of mitochondria and autophagy. Measurement of ATPase activity also suggested a decrease of mitochondrial energy metabolism in ADR-treated myocardium (Figure 3C). The autophagy marker LC3B, cell apoptosis enzyme cleaved caspase3, autophagy regulatory enzyme Beclin 1, and ER-stress-related transcription factor CHOP had all been increased in ADR-treated heart tissues (Figure 3D).

### Exogenous increasing miR-432-5p improves ADR-induced toxicity *in vivo*

**Figure 4:**
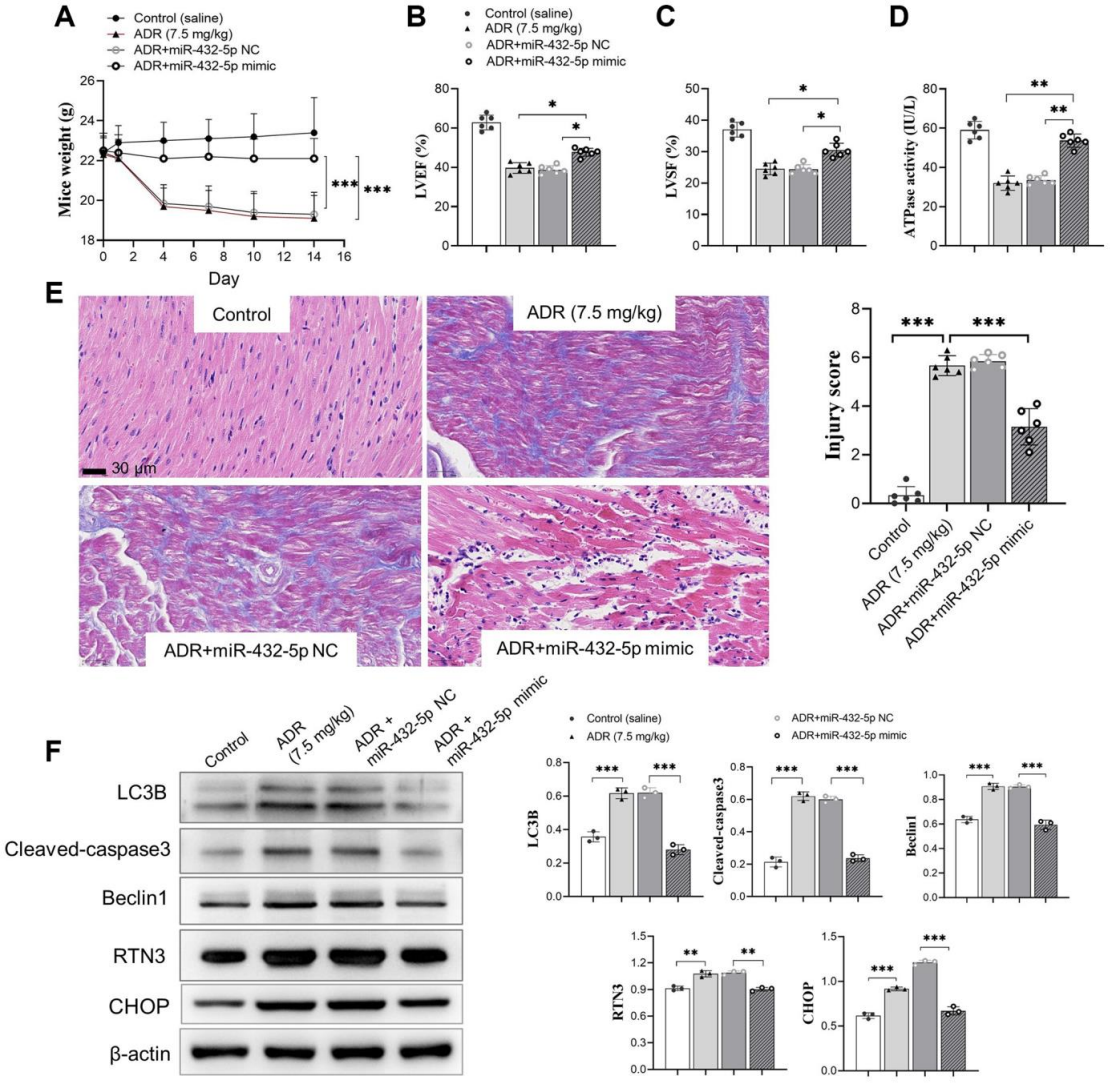
**miR-432-5p alleviates ADR-induced toxicity in mice.** (**A**) The body weight change after ADR-treated mice received miR-432-5p; (**B**) The LVEF improves after miR-432-5p treatment; (**C**) Improved LVSF after miR-432-5p treatment; (**D**) The activity of ATP synthesis in mouse myocardial tissue; The immunohistochemical images (**E**). Results showed the injury caused by ADR was improved by miR-432-5p; (**F**) The protein expressions of LC3B, cleaved-caspase 3, Beclin 1, RTN3, and CHOP decreased in ADR stimulated cells pretreated by miR-432-5p. ^*^p < 0.05, ^**^p < 0.01, ^***^p < 0.001.

The exogenous miR-432-5p was introduced in ADR-stimulated myocardium. Upregulation of miR-432-5p improves the decreased body weight (Figure 4A) accompanied by improved LVEF, LVSF and activity of ATP synthesis (Figure 4B–4D) in ADR-treated mice. The immunohistochemical staining demonstrated that the injury caused by ADR was improved by miR-432-5p (Figure 4E). Autophagy is activated in cells that receive various stimuli (or stresses). LC3 (microtubule-associated-proteinlight-chain-3) forms autophagosome-encapsulated degradation products and is transported to lysosomes for degradation. LC3B is an LC3 isotype and a marker of myocardial tissue damage [21]. Cleaved caspase3 and Beclin 1 are important apoptosis marker of cells [22, 23]. We measured the above important markers in tissues. Exogenous increasing miR-432-5p in ADR-treated mice decreased protein expression of LC3B, cleaved caspase3, Beclin 1, and RTN3 (Figure 4F), which suggested that exogenous increasing miR-432-5p could reduce ER stress, apoptosis and autophagy.

### miR-432-5p attenuates ADR-induced toxicity via RTN3

**Figure 5:**
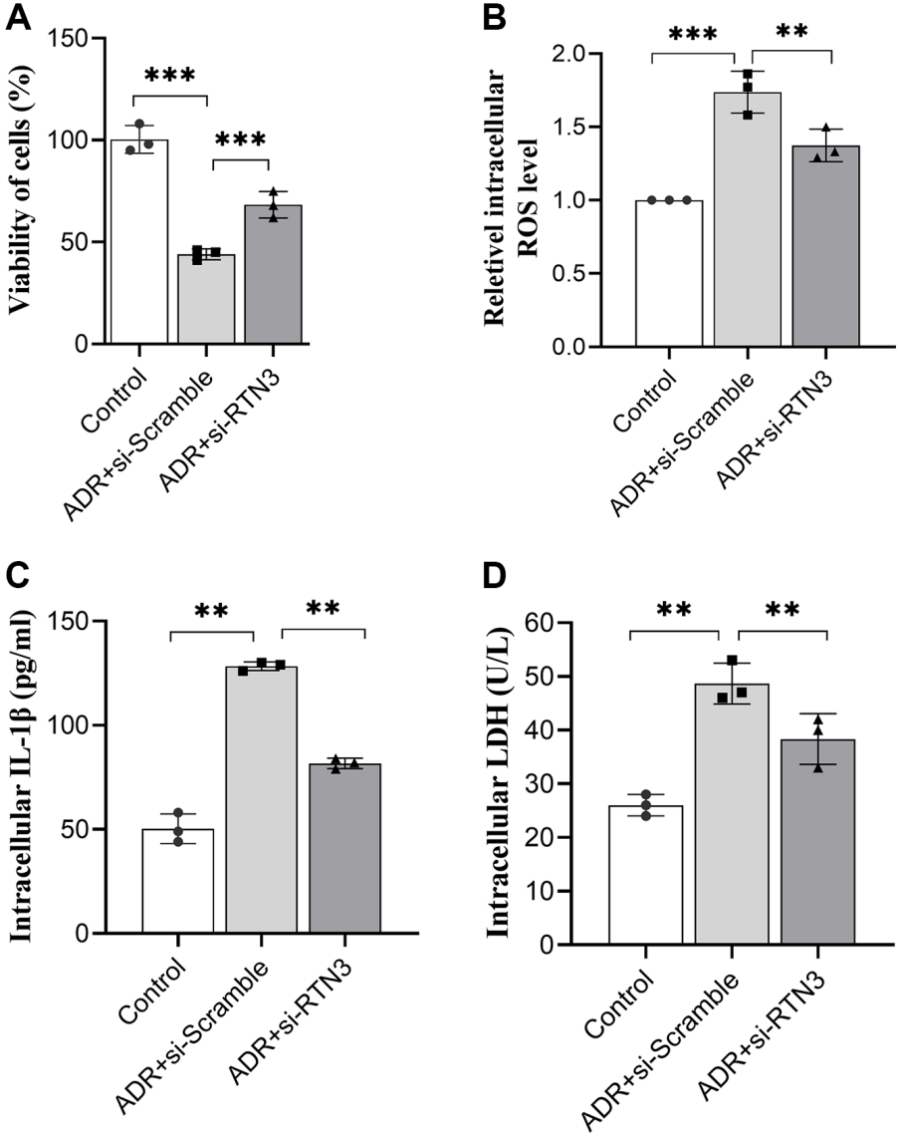
Silencing RTN3 attenuates ADR-induced toxicity to primary cardiomyocytes by improved cell viability (**A**), ROS (**B**), IL-1β (**C**), and LDH (**D**) levels.

**Figure 6:**
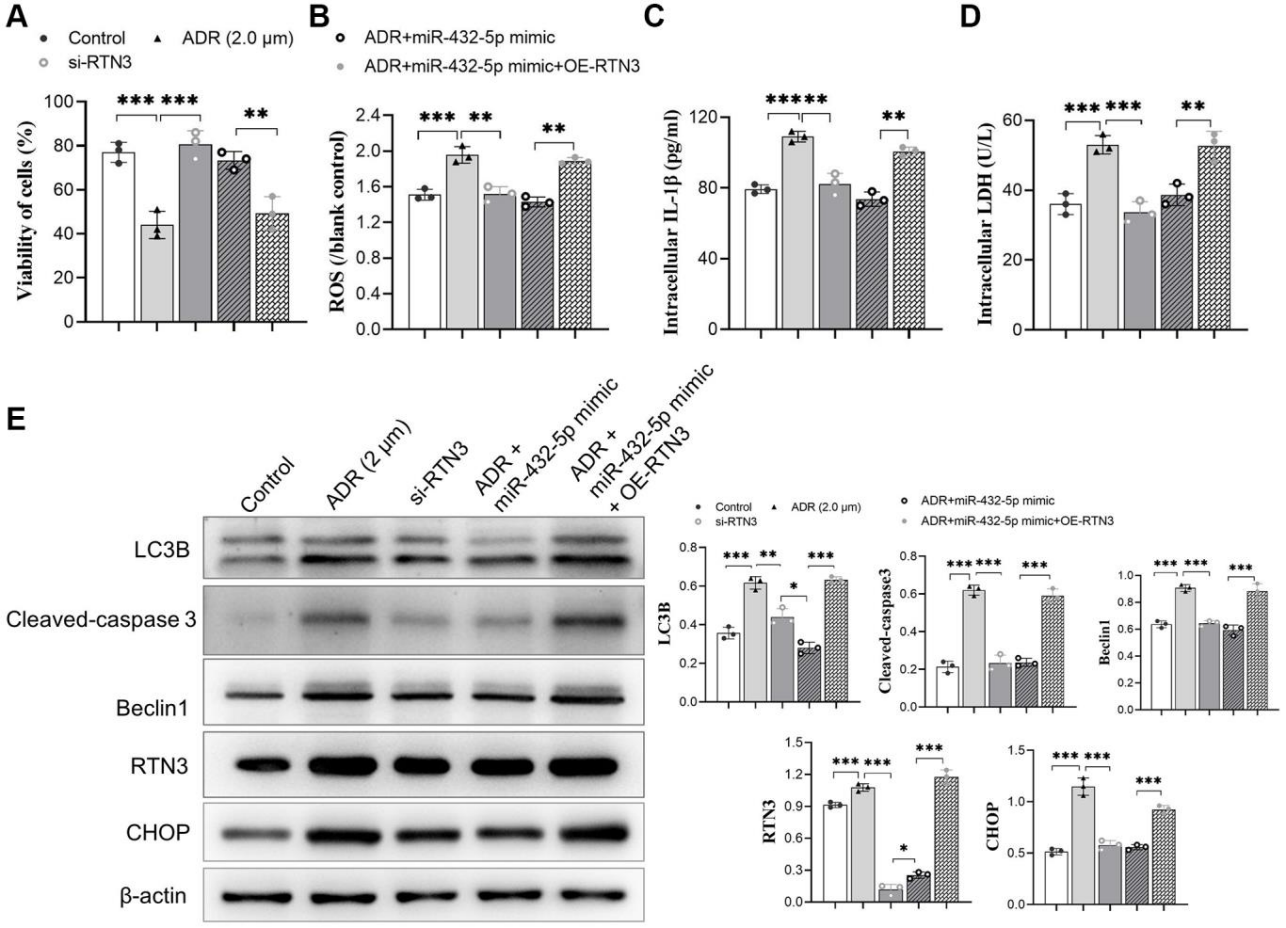
**miR-432-5p attenuates ADR-induced toxicity via RTN3.** (**A**–**C**) The mimic of miR-432-5p, si-RTN3, or OE-RTN3 were transfected into cardiomyocyte, and si-RTN3 and miR-432-5p mimic attenuate ADR-induced toxicity by cell viability (**A**), ROS (**B**), IL-1β (**C**) and LDH (**D**) level with the protection that was abolished by OE-RTN3; (**E**) Western blot analysis indicates RTN3 was knockdown or upregulated and accompanied by changing of LC3B, cleaved-caspase 3, Beclin 1, and CHOP in cardiomyocyte. ^*^p < 0.05, ^**^p < 0.01, ^***^p < 0.001.

To verify the detrimental role of RTN3 on ADR-induced heart injury, we silenced RTN3 in the primary cardiomyocytes. Compared with scramble siRNA control, knocking down RTN3 significantly increased cell viability, and reduced the production of ROS and LDH, which demonstrated that RTN3 significantly contributed to the ADR-induced toxicity to cardiomyocytes (Figure 5A–5D). Next step, we verified whether the effect of miR-432-5p decreasing ADR induced toxicity is relying on RTN3. Compared with cardiomyocytes treated by ADR, si-RNT3 and the miR-432-5p mimic attenuate ADR-induced toxicity while could not attenuate the toxicity when RTN3 was over-expressed (Figure 6A–6D); which was confirmed by western blot analysis when RTN3 was upregulated along with partial upregulated expression of LC3B, cleaved-caspase3, Beclin1 and CHOP in cardiomyocyte (Figure 6E).

## DISCUSSION

In the current study, we found miR-432-5p, a miRNA that protects myocardial infarction [20] via binding with RTN3 to attenuate ADR-induced toxicity. The downregulation of RTN3 by miR-432-5p improves the myocardium function by enhancing mitochondrial ATP synthesis, reducing oxidative stress and inflammation damage to the endoplasmic reticulum, and reducing cell apoptosis. ADR is an inhibitor of the DNA topoisomerase II enzyme and is commonly used for the treatment of various tumors. However, the cardiac toxicity of ADR severely restricts its clinical use, including various types of arrhythmias, pericardial and myocarditis, acute hypertension, congestive heart failure, and even death [24]. The pathological mechanism of ADR-induced cardiotoxicity is complex, including mitochondrial damage [25], oxidative stress [26], calcium dysregulation [27], and myocardial cell apoptosis [28]. These mechanisms of action interact with each other, ultimately leading to structural damage to myocardial cells and causing cardiac dysfunction, that is, loss of myocardial contractility.

The ER maintains the stability of the intracellular environment, and ERs induced by physiological or pathological factors have a protective effect on cells or cause cell apoptosis. ERs are closely related to the occurrence and development of atherosclerosis, ischemic cardiomyopathy, essential hypertension (hypertension), and heart failure [29–32]. The mechanism of ADR-induced cardiotoxicity is not yet clear, and current research evidence shows that oxidative stress, autophagy, mitochondrial damage, and cell apoptosis are the most common mechanisms [33]. The occurrence of this process is temporal, with oxidative stress leading to mitochondrial dysfunction, endoplasmic reticulum stress, and autophagy, activating a series of pathways, and causing myocardial cell apoptosis [34]. In the current study, the mitochondria protection effect of miR-432-5p was confirmed by increasing cell viability and reducing inflammation and oxidation. Mitochondria play an important role in energy metabolism as the engine of life. Mitochondria are involved in oxygen-free radicals’ production, calcium homeostasis maintenance, cell apoptosis, aging, and tumors. A recent study [35] reported that doxorubicin can cause cardiomyocyte apoptosis, increase intracellular ROS production, and reduce intracellular ATP concentration and mitochondrial membrane potential in cardiomyocytes. Our analysis also revealed that mitochondrial ATP synthesis significantly decreases in ADR-treated myocardium. We speculate ADR first causes enhanced cellular oxidative stress, increased cellular oxidative stress leads to mitochondrial damage, further leading to structural changes in cells and tissues. Which is reflected in the results of pathological analysis, structurally, ADR causes disordered arrangement of muscle fibers, interstitial edema, and increased collagen between muscle fibers and muscle bundles in some areas. Mitochondrial gene expression alteration is the direct cause of myocardial damage caused by drugs [36]. Compared with nuclear genomic DNA, mitochondrial DNA has the characteristics of lacking protection from introns and histones, being closer to endogenous oxygen radicals, and imperfect repair ability, making it more susceptible to damage from reactive oxygen species (ROS) and other genotoxicity, leading to abnormal changes in mitochondrial DNA [37].

Due to mitochondrial dysfunction, ATP content typically decreases by more than 30%, and substrate utilization is disrupted to optimize oxygen utilization (known as metabolic modeling) and manifested as a relative increase in glycolysis and a decrease or maintenance of fatty acids [38]. The ER, as a neighboring organelle of mitochondria, cooperates with mitochondria to coordinate calcium kinetics and energy generation. We also observe that autophagy activation caused by ER stress also plays an important role in the process of ADR-induced toxicity. Autophagy is a metabolic process in which cells degrade damaged or excess cellular components through lysosomes or vacuoles (yeast) to produce energy or small biological molecules. It is highly conserved in eukaryotes. The ER autophagy is a newly identified form of autophagy and is a selective form of autophagy that ensures the timely removal of damaged ER, thereby protecting cells from damage caused by excessive ER stress. ER autophagy is driven by the synergistic or individual activation of a large number of ER autophagy receptors embedded or recruited onto the ER membrane. The ROS, inflammatory activation, and mitochondrial dysfunction lead to a series of interactive damage processes. When abnormal proteins, protein complexes, or individual organelles (such as mitochondria or ER) are damaged, a large number of autophagy and autophagy receptor-mediated programs are activated. The ER part is fragmented and delivered to the lysosome/vacuole compartment for clearance, which is used to digest cytoplasmic contents. This process mainly drives the rapid mobilization of proteins, membranes, and sugars to generate components through three pathways: ER macroautophagy, ER microautophagy, and LC3-dependent vesicles, regulating the size, shape, and activity of the main biosynthetic organelles in cells [39]. LC3B is one of the four LC3 homophones and one of the markers of ER autophagy [21, 40]. In the current study, we confirm that miR-432-5p reduces ER stress and ER autophagy by decreasing the RTN3 protein expression.

During the process of apoptosis, caspases are cleaved and activated. Subsequently, they lead to the cleavage of hundreds of proteins, and promote cell disintegration. Cysteine proteases are expressed as inactive zymogens and are activated in response to apoptotic stimuli through dimerization or specific protein hydrolysis cleavage. Activated cysteine protease cleaves and activates other family members and several key proteins [41]. Caspases-3 is an important apoptotic effector of cysteine proteases and is also a “switch” that regulates ERs. Autophagy may enhance the clearance of apoptotic and necrotic cells to promote the repair of damaged tissue [42]. Beclin l is a multifunctional protein that plays an important role in cell differentiation, apoptosis, and autophagy, promoting autophagy in many cell types. Our analysis shows that ADR stimulation also causes increasing in Beclin 1. This indicates that ADR stimulation activates both autophagy and apoptosis, while Zhu [43] reported caspase-3 cleavage Beclin 1 to inactivate autophagy and promotes apoptosis. We speculate that this may be due to the fact that the ADR toxicity response we induced in the model belongs to the early stage of the disease, while the study reported by Zhu et al. may occur in the later stage, where the increased stimulation of Beclin 1 further activates caspase-3 and augments apoptosis. In the current study, the RTN3 was demonstrated to aggravate ER stress, and consequently induced ER autophagy and apoptosis, which provides a fundamental mechanism for miR-432-5p.

In conclusion, our study showed that the stimulation of ADR leads to impaired mitochondrial function, leading to endoplasmic reticulum stress and apoptosis. Meanwhile, ADR stimulation causes the expression level of miR-432-5p to reduce significantly, activating the endoplasmic reticulum stress RTN3 pathway, leading to vascular endothelial damage, and affecting myocardial cell metabolism. Specifically, in the toxic response caused by ADR, it first causes a decrease in ATP synthesis in myocardial cells, stimulates upregulation of endoplasmic reticulum autophagy-related proteins LC3B and Beclin 1, and leads to activation of apoptotic protein caspase-3. Exogenous enhancement of miR-432-5p can attenuate the toxic response of ADR in myocardial tissue, but the effect still needs further confirmation *in vivo* research.
